# The Treatment of Dental Caries in Children*NOTE.—We reprint this article because it is so diametrically opposed to generally accepted practice. There is much in it, however, that needs careful consideration. The article seems radical and even falacious, but in reading remember it is written with septic conditions in mind. If root canals in deciduous teeth can’t be made aseptic it were better to sacrifice the teeth.—Editor.

**Published:** 1914-06-15

**Authors:** F. St. J. Steadman

**Affiliations:** London, Eng.


### THE TREATMENT OF DENTAL CARIES IN CHILDREN*.

BY F. ST. J. STEADMAN, L. D. S., LONDON, ENG.

A clean, healthy, functional mouth is of vital importance to the health of the growing child. The health of our children is essential not only to their own happiness and welfare, but to our happiness also. Further, the vitality and prosperity of the nation must depend largely upon the health of our men and women. To attain the maximum amount of efficiency, either mentally or physically, it is essential that those important years during which growth of the mind

*NOTE.—We reprint this article because it is so diametrically opposed to generally accepted practice. There is much in it, however, that needs careful consideration. The article seems radical and even falacious, but in reading remember it is written with septic conditions in mind. If root canals in deciduous teeth can't be made aseptic it were better to sacrifice the teeth.—EDITOR.

and body is taking place should be as free as possible from disease, *especially from chronic disease, the effect of which spreads over a period of years.* If we allow a child to grow up with an unhealthy mouth, there is ample evidence to show that its growth will suffer—in very many cases severely. I am of opinion, based on careful and extensive observation, that it will never afterwards make up the ground it has lost.

In order to prevent dental disease, it is essential that the mouth be functional; by this I mean broad, well-developed arches with the teeth in their proper occlusion. There must be an entire absence of tender teeth or gums; one tender tooth may render functionless the whole of one side of the mouth. This result can be obtained by (1) breast-feeding in infancy, (2) nasal breathing, and (3) a proper diet. J. F. Colyer has shown that breast-fed children have slightly broader and better-formed arches than artificially-fed children. Without proper nasal breathing a functional healthy mouth is an impossibility. I would remind you that mouth-breathing in the early years can be diagnosed by the persistent marginal gingivitis around the incisor teeth, and later by the effect the nasal obstruction, the usual cause of the mouth-breathing, has upon the maxillary bones, viz., the narrow, high V-shaped arch often associated with superior protrusion, or outstanding canines; and later still by the stupid look and pigeon-shaped chest which these unfortunate children eventually develop.

The diet should be carefully attended to. When soft, sticky carbohydrate food has been eaten, it must be followed by some detergent food, such as an apple or other fresh acid fruit. This mechanically cleanses away the soft films of carbohydrate food which remains clinging to the crowns of the teeth, especially in pits and deep fissures. The acid fruits are best because they not only cleanse the teeth better but they stimulate the flow of saliva. Sweets between meals, and biscuits or such-like food last thing at night, should be absolutely forbidden. If these rules be strictly carried out, a tooth-brush is unnecessary, and with the poor should be avoided. A tooth-brush among them, on account of the unclean condition in which it is very frequently kept, often does more harm than good. Indeed, it is not uncommon to find one "family" brush used to "clean" the teeth of the whole family, and in at least one case I found it was being used for other small household jobs as well.

I endeavour to educate the parents to bring their children for regular periodical inspection, so that caries can be treated early. *The importance of treating caries early before infection of the pulp occurs can hardly be exaggerated.* Unfortunately, this ideal, as you well know, is only too frequently not carried out, and children are brought to us with several teeth showing advanced caries. Too often parents pay no attention whatever to the condition of their children's teeth until they complain of pain.

All deciduous teeth in which the decay is sufficiently advanced to infect the pulp I extract, and their antagonists also. I only fill these teeth when I am quite sure that the pulp is not infected. This condemns at once the vast majority of decayed deciduous teeth because the pulps of these teeth are relatively larger than in permanent teeth, so that a comparatively small cavity is sufficient to infect the pulp. By infected pulp I do not necessarily mean an exposed pulp; many pulps are infected long before actual exposure has occurred, as shown by pain and tenderness in the tooth and very frequently by enlarged and tender submaxillary lymphatic glands. These glands are so frequently involved in children that I now make it a routine practice to carefully examine for them. I would remind you that the lymphatics from the lower incisor teeth, and the gums around, drain into the submental glands. The efferent vessels from this group pass partly into the submaxillary group and partly to a deep cervical gland situated on the superficial surface of the internal jugular vein at the level of the cricoid cartilage. The lymphatics draining the regions of the lower canines, premolars and molars pass into the submaxillary group of glands, the efferent vessels from which pass into the upper deep cervical group situated along the anterior border of the sternomastoid. The lymphatics draining the upper teeth and gums, following the course of the internal maxillary vessels, pass into the parotid glands, the efferent vessels of which pass into the upper deep cervical group. The glands under the mandible are best palpated from the front of the patient, whose head should be bent slightly forward. The cervical glands are best palpated from behind with the patient's head in the same position as before. It is essential to see that all clothing is removed from around the neck before commencing the examination.

I treat the permanent molar teeth in the same way as the deciduous teeth if the roots are incomplete at the time of the pulp infection. Why should a tooth with an infected pulp be removed? The reason is that an infected pulp is a source of greater danger to the child. It is in my opinion quite impossible to devitalize and remove it and fill the root canal as we do in permanent teeth because, unless a skiagram be taken, which is not practicable in the majority of cases, it is impossible to be sure whether the apex of the tooth is complete or not. The times when the various roots are complete given in the text-books are merely approximate, and vary so greatly in different children that for all practical purposes they are too unreliable to base treatment upon. This being the case, the treatment recommended by many practitioners amounts merely to this—that the pulp is sterilized "as far as possible," capped, and the filling inserted over it. This proceeding is exceedingly dangerous. In the majority of cases so treated the pulp eventually suppurates and dies under the filling. The infection now readily spreads through the widely open apex of the root to the nearest lymphatic glands. These infected glands are very dangerous. It is probable that tubercle bacilli not infrequently gain entrance into the body in this way. It is certainly a fact that glands infected from septic teeth do not always clear up and return to their normal state after the offending tooth has been shed or extracted. It is also a fact that these glands sometimes become very much smaller after the tooth has been lost but never *quite* disappear, and break out again many years later; frequently when the child reaches the age of puberty he is found to be definitely tubercular. In such cases it seems reasonable to suppose that the tubercle bacilli first gained entrance through the septic tooth and remained dormant for a time, only to break out again years after, when, for some cause or other, the child's power of resistance is temporarily lowered.

Occasionally these infected glands break down and the infection is carried to other parts of the body, giving rise possibly to tubercular lesions in the bones, or it is even possible that tubercular meningitis may be set up. Even if this extremely serious, or I should say disastrous, result does not often take place, infected glands in the neck, whether tubercular or not, with all the attendant ill-effects on the health of the child, is far too great a price to pay for the retention for a few years longer of a tooth. Enough has been said on this point to show that the dental surgeon who saves teeth with infected pulps, which he cannot make healthy, is undertaking a very grave responsibility indeed.

Apart, however, from the infection of the lymphatic glands, septic teeth have a most profound adverse influence upon the general health of the child. Every time a child with septic teeth eats, it wipes out the septic matter in these teeth and conveys it to its stomach with the food. The result is that it frequently suffers from gastro-intestinal infection, and, consequently, the child does not develop properly either mentally or physically, as we shall see later on when considering the question of mastication.

But it may be asked, granted that all teeth which cannot be made perfectly healthy should be removed, why is it necessary to remove their antagonists, which may be quite healthy and sound?

In answering this question, let us take for example a typical case, only too frequently seen, where a child has the four lower deciduous molars unsavable, and the four upper molars sound and healthy. If these four septic lower teeth are extracted we leave the four upper teeth useless, that is to say, functionless. For these teeth have three main functions to perform: (1) The mastication of food; (2) to assist speech; and (3) for beauty. It is clear that these four upper teeth cannot possibly take part in the mastication of food, because their antagonists have been lost. The child having learnt to speak can, as experience shows, continue to do so quite well, even if all its teeth are extracted. The question of beauty is unimportant, indeed hardly arises, because these teeth will be replaced by their permanent successors in due course. Experience proves that teeth which are functionless quickly come to grief, becaause they are no longer mechanically cleansed during the process of mastication of the harder foodstuffs. The result of this is that not only do they quickly become decayed, but the gum around them becomes inflamed and unhealthy. Consequently the health of the child, which has improved owing to the extraction of the septic lower teeth, very soon begins to suffer again.

An experiment carried out by Mr. J. F. Colyer may make this point clear. A young child who had one lower molar tooth missing, but all the rest of the teeth present and healthy was given a meal of soft food (bread and butter), and was then given an apple. The eating of the apple mechanically cleansed away all the soft food which remained clinging round the teeth after the meal of bread and butter, *except around the tooth which was opposite the place where the tooth was missing.*

Around this tooth was a thin film of soft carbohydrate food, and when this was scraped off a little ring of inflamed gum all around the tooth was seen, due to the frequent lodgment of food.

Now this experiment clearly demonstrates that the proper treatment was to remove this functionless tooth, because it is an established fact that this film of soft carbohydrate food, if left upon it, becomes acid and starts the process of decay, and moreover this small area of inflammation of the gum may spread around the whole mouth, because the gum at this point is tender, so that the child avoids biting on that part of the mouth; consequently several normal functional teeth may be rendered useless and become septic also.

These easily demonstrable facts clearly show that a functional mouth is a self-cleansing mouth; and that in a mouth in which there are functionless teeth there will be areas in which food will stagnate and decompose, causing caries of the teeth and inflammation of the gums; further, that this inflammation being tender will prevent mastication and so render the whole mouth functionless. From this it follows that not only should septic teeth, which cannot be made healthy, be removed, but their antagonists too. The maxim just laid down may, in certain cases, happily not common, lead one on to perform a very drastic operation, viz., the removal of all the teeth; nor do I hesitate to perform this operation, the logical outcome of my reasoning, where I consider it necessary. I am quite convinced that on several occasions this operation may definitely be said to have saved the life of the child. In other cases an unhealthy, weakly child has been made healthy.

There is a certain "bugbear" raised by some dental surgeons against this line of treatment which must now be considered. They say: (1) That extensive extraction of the teeth in a young child prevents the proper growth of the jaws; and (2) that it later on causes overcrowding and irregularities of the teeth.

Let us consider these statements.

If the extraction of teeth adversely influenced the growth of the jaws, it is clear that if several teeth are removed on one side and not on the other, the jaws on the side from which these teeth have been extracted will show a deficient growth, whereas the side where the teeth had not been removed would grow normally, giving the whole face a lopsided appearance. But experience shows that nothing of the kind happens; on the contrary, the jaws still reach their normal development on both sides in spite of the loss of teeth on one side.

Or again, if we extract teeth from the upper jaw and not in the lower, the upper jaw will not grow properly, but the lower will, so that we shall have brought about that ugly condition known as "inferior protrusion." Nothing of the kind happens; showing that the growth of the upper jaw has not in any way been affected by the extraction of the teeth.

We see then that the first statement does not bear thoughtful examination.

With regard to the second, it will depend largely upon the age of the child at the time of the operation. If the first permanent molars have fully erupted and are therefore properly antagonizing each other, the cusps of the one fit into the cusps of the other and prevent any marked forward movement taking place, and as we have already seen, the jaws grow normally, so that there will be the normal sufficient space left for the eruption of the teeth which are to succeed those lost; the result will be therefore that the teeth are not overcrowded and no irregularity in their arrangement will occur.

If, however, the teeth are extracted in a very young child before the first permanent molars have become locked, they will undoubtedly come too far forward and encroach upon the space which should be occupied by the premolars, and overcrowding and irregularity will occur. This, however, can easily be remedied by the extraction of four permanent premolars, one at each corner of the mouth, as soon as they begin to erupt. The final result of a case so treated is that all the teeth are in proper occlusion. There will be no space, but the child will be four teeth short. The real effect from the dental point of view is absolutely *nil.* The child will never feel the want of the missing teeth, but, on the other hand, it will have become strong, healthy, and full-grown, instead of weakly and delicate. Surely such a result is cheap indeed at the price.

There is, however, another "bugbear" which has to be met. We are asked, in this case more especially, by some medical practitioners: What about mastication? Surely, if you extract so many teeth the child will suffer because it is unable to masticate its food?

Let us reflect for a moment what the process of mastication means and for what is its purpose. Mastication is the first stage of the breaking up and preparation of the food for its assimilation. The food as it is taken in at the mouth cannot be assimilated by the body. Unless it undergoes certain changes it will pass right throough the body without nourishing it and the individual will die of starvation. The factors which bring about these changes in the food are very numerous, and mastication is one of the earliest it comes across, and, as I think we shall presently see, one of the least important in these days of modern civilization in which we eat soft and well-cooked food, capable of easy assimilation. The point which we have to consider is, can we do away, in the young child, with this process of mastication, and rely upon the remaining factors which bring about these necessary changes in the food for its proper assimilation?

In order to answer this question we have simply to ascertain whether the food the child is taking is nourishing it or not. If it is the child will have a healthy appearance. It will have "rosy" cheeks and bright eyes. It will be intelligent and it will be well grown physically, that is to say, it will be of the normal body-weight for its age and size.

If the food is not nourishing it the child will be pale or "pasty" faced, and will be mentally and physically ill-developed. It will be under weight. In other words, it will have a half-starved appearance. Mr. J. F. Colyer has taken a number of children who have this half-starved appearance, children who were obviously under the normal weight for their size and age, and has weighed them before and after the removal of a large number of septic teeth, on the lines laid down above, and has found that they not only rapidly *regain their normal weight*, but, much more even than this, they rapidly lose their unhealthy appearance and become rosy-cheeked, and, moreover, a child backward at school will often quickly reach its normal standard. I have myself weighed a large number of these children before and after treatment and have been able to amply confirm Mr. Colyer's results. Recently the father of one of my young patients at the Belgrave Hospital for Children took the trouble to find out where I lived, to visit me for the special purpose of thanking me for my treatment of his son, aged 9. Some fourteen months before I had removed twelve teeth. The father told me that up to that time he had always been delicate and ailing, he had had many restless nights from toothache, and had constantly needed medical care. He was small, under weight, pale faced, and very backward at school. He had "suffered terribly." A few days after the operation he began to change rapidly. He has now rosy cheeks, looks well, has regained his normal weight, has not once been ill, and has made rapid strides at school. It seemed, as his father said, as though a millstone had been removed from about his neck. Septic teeth do form a millstone in very truth, a millstone which checks the normal development of the child, both mentally and physically, and undermines the physique of the future man or woman.

Another case which came under my care a year ago illustrates many points which I have been trying to make this evening and is therefore worth mentioning in some detail. The patient was a boy, aged 5, and was brought to me by his mother on account of pain in his teeth which caused him to have restless nights. On examination I found that he had had no less than eleven amalgam fillings inserted in his deciduous molars. Three of these teeth were obviously "dead" and tender. Pus could be squeezed out from pockets around these three dead teeth. The gums all around the mouth were inflamed and dirty. *It was clear that the child had not used his teeth for the purpose of mastication for some time.* The submaxillary lymphatic glands on each side were swollen and tender, as also were the upper deep cervical glands on the left side.

The mother told me that the child never seemed well, that he was very irritable and always cried himself to sleep when put to bed, and frequently woke during the night, at such times complaining of pain in his teeth. "That he had not had a good night's rest for months," and "that he did not care for his food, and did not eat enough." The child's general appearance amply bore out the mother's statement. He was small for his age and pale faced; his eyes lacked the brightness and intelligence of the normal child.

The dentist who filled the teeth had told the mother that nothing further could be done until the child was 10 or 11 when these teeth would be shed. He said that it was absolutely necessary to retain these teeth until then for the purpose of mastication. A second dentist had confirmed this opinion. In face of these opinions of two of my fellow practitioners I had some difficulty in getting the parents' consent to the immediate extraction of all the deciduous molars. Fortunately, I was supported on this occasion by the family doctor (more often than not I regret to state that I have found in him a powerful opponent). The operation was performed on March 18, 1913. The effect was startling. On that night, and each following night, the child slept well. There was no crying out and no restlessness whatever. On April 22 the glands were only just palpable and were no longer tender. The beneficial effect on the child's health of the good nights was obvious. The pallor of the cheeks and sleepy, tired look had vanished. The gums and mouth were clean and healthy. The mother said that the improvement in health "seemed marvellous. The child was altogether brighter in himself. He was eating well and seemed always hungry." This child was not weighed, but it was apparent that he was now rapidly putting on weight. The early extraction in this case will, I fear, lead to forward movement of the first permanent molars and crowding later on, which can be treated as I have already indicated. This I have pointed out to the parents, who seem to think that this is not worth considering in face of the immense improvement in the child's mental and physical growth—an attitude I think very intelligent person will heartily endorse.

Supposing thirty children, otherwise healthy, are arranged in a group. Ten of these children have all of their teeth present, and these teeth, together with their mouths, are perfectly healthy; ten more have had most, if not all, their teeth removed, but their mouths are otherwise healthy; and the remaining ten have septic teeth and dirty, inflamed mouths. It is perfectly easy for the trained eye to pick out these last ten children from their miserable unhealthy appearance, without any examination of their mouths. But with regard to the remaining twenty it is quite impossible to pick out from them those children who have had the large number of extractions. To all appearance, as far as the eye can tell, they are as healthy as the remainder. Moreover, if they are now weighed it will still be impossible to pick them out.

But there is another aspect to this question of mastication which must not be forgotten, and which is well shown in the second case just quoted above. No child with septic tender teeth in its mouth will masticate properly, because the process is painful. Therefore the function of mastication has already been destroyed before extraction of the teeth. Moreover, it frequently happens that by removing tender carious, deciduous molars the area of mastication is actually increased by restoring to usefulness the permanent six-year-old molars behind them.

In the face of these facts it is of no use saying that mastication is *essential* to the well-being of the child, that the food can not be properly assimilated without it; for the test is the child's mental and physical growth, and it stands this test.

It is, of course, preferable to have the mouth healthy and with the normal number of *clean and healthy teeth.* It has been shown conclusively, however, that it is infinitely better that the child should have no teeth at all rather than septic unhealthy ones.

The sooner these important facts are realized the better it will be for the health of the children, and from this it follows as the night the day, the better it will be for the health of the nation—*British Dental Journal, May, 1914.*

